# Engineering of Bispecific Affinity Proteins with High Affinity for ERBB2 and Adaptable Binding to Albumin

**DOI:** 10.1371/journal.pone.0103094

**Published:** 2014-08-04

**Authors:** Johan Nilvebrant, Mikael Åstrand, Maria Georgieva-Kotseva, Mattias Björnmalm, John Löfblom, Sophia Hober

**Affiliations:** Division of Protein Technology, School of Biotechnology, KTH Royal Institute of Technology, Stockholm, Sweden; Deutsches Krebsforschungszentrum, Germany

## Abstract

The epidermal growth factor receptor 2, ERBB2, is a well-validated target for cancer diagnostics and therapy. Recent studies suggest that the over-expression of this receptor in various cancers might also be exploited for antibody-based payload delivery, e.g. antibody drug conjugates. In such strategies, the full-length antibody format is probably not required for therapeutic effect and smaller tumor-specific affinity proteins might be an alternative. However, small proteins and peptides generally suffer from fast excretion through the kidneys, and thereby require frequent administration in order to maintain a therapeutic concentration. In an attempt aimed at combining ERBB2-targeting with antibody-like pharmacokinetic properties in a small protein format, we have engineered bispecific ERBB2-binding proteins that are based on a small albumin-binding domain. Phage display selection against ERBB2 was used for identification of a lead candidate, followed by affinity maturation using second-generation libraries. Cell surface display and flow-cytometric sorting allowed stringent selection of top candidates from pools pre-enriched by phage display. Several affinity-matured molecules were shown to bind human ERBB2 with sub-nanomolar affinity while retaining the interaction with human serum albumin. Moreover, parallel selections against ERBB2 in the presence of human serum albumin identified several amino acid substitutions that dramatically modulate the albumin affinity, which could provide a convenient means to control the pharmacokinetics. The new affinity proteins competed for ERBB2-binding with the monoclonal antibody trastuzumab and recognized the native receptor on a human cancer cell line. Hence, high affinity tumor targeting and tunable albumin binding were combined in one small adaptable protein.

## Introduction

Numerous studies have demonstrated that smaller affinity proteins can be engineered for high affinity and specificity and are thus expected to be excellent alternatives to antibodies for clinical applications. The smallest affinity proteins also have the option to be produced by chemical peptide synthesis, which further extends the possibilities of modifications and conjugations. Several non-immunoglobulin binding proteins have been generated against different biomarkers, for example designed ankyrin repeat proteins [1], [2] and Affibody molecules [3], [4]. Such small binding proteins have shown quick biodistribution, good penetration into tumor tissue and fast elimination from serum and non-diseased tissues [5], [6]. Small size is generally attractive for therapy, while a longer serum half-life is usually necessary to extend the exposure time of a drug and to limit the dosing frequency. Both experiments and modeling approaches have identified two groups of affinity reagents with a high potential for solid tumor targeting [7]–[9]. The first group constitutes very small molecules with a high affinity for the tumor target, but generally with short half-lives. The second group includes long-lived molecules with a size above the renal filtration threshold, such as antibodies. To further improve the potency of molecules that belong to the first group, such as peptides and small proteins, several strategies have been explored to extend their *in vivo* half-life [10] One promising approach utilizes association to albumin in order to exploit the favorable pharmacokinetics of this abundant molecule [10]–[12]. With this strategy, the protein may also take advantage of the biological regulation performed by the neonatal Fc-receptor (FcRn), which binds albumin and can rescue it from lysosomal degradation [13]. Albumin-binding or fusion may also reduce undesired accumulation in healthy organs (e.g. in the kidneys) of the targeting molecule and its potential payload, which can otherwise be dose-limiting in therapeutic applications [14]. However, present strategies for extending the half-life of therapeutic proteins also result in an increased size, for example by (i) fusion to a long-lived serum protein, (ii) fusion to a serum-protein binding partner or, (iii) nonspecifically through PEGylation. Thus, these approaches counteract the advantage of having a small size.

Combining small size with long serum half-life would be very attractive. By endowing a small target-specific binding protein with albumin affinity, or as in our strategy, by engineering target binding directly into a small albumin-binding protein, renal elimination could possibly be avoided. As a first step to attain a miniaturized targeting molecule with an extended circulation time, we have previously generated target-binding molecules based on a small albumin-binding domain (ABD) derived from streptococcal protein G [15], [16]. These novel target-binding molecules are here referred to as ABD-Derived Affinity ProTeins (ADAPTs). By diversifying a surface of the domain that is not directly involved in albumin binding, molecules can be selected to bind a novel target and still retain their ability to bind albumin. This strategy has been used to select binders to a number of proteins and recently it was applied to the cancer-related epidermal growth factor receptor 3 [17].

Herein, we describe the engineering of high affinity binders to the human epidermal growth factor receptor 2 (HER2 or ERBB2) with retained ability to associate strongly to albumin. Over-expression of ERBB2 is observed in 20–30% of metastasizing breast cancers, and this protein is a well-established diagnostic marker and also a therapeutic target. It has been linked to other forms of cancer and it is associated with poor prognosis and resistance to certain therapies, hence it is also used as a prognostic tool [18], [19]. Therefore, it is of great interest to develop novel prognostic and therapeutic molecules with affinity to ERBB2. Here, different selection- and directed mutagenesis strategies were combined to achieve binding molecules with high affinity. First, phage display was used to select a first generation ADAPT that was able to bind ERBB2. Affinity maturation libraries were thereafter designed based on alanine scanning data of the ERBB2-binding surface. During affinity maturation, improved ERBB2-binders were isolated using successive phage- and bacterial display selections. These novel ADAPTs were shown to bind strongly to the native receptor expressed on SKOV3 cells by recognizing an epitope that overlapped with the binding site of trastuzumab. The albumin binding affinity could be modulated by single substitutions in non-ERBB2-binding regions that were identified during the selections, which may enable future fine-tuning of pharmacokinetic profiles. The methods and results presented here illustrate new ways of engineering multifunctional proteins and indicate a potential for this recent class of agents aimed at tumor targeting.

## Materials and Methods

### DNA sequencing and cloning

DNA encoding ADAPTs from individual colonies isolated after selection were PCR-amplified, sequenced and sub-cloned from the phagemid or cell display vector, respectively, to a common expression vector as described earlier [16].

Variants of ADAPT_ERBB2-1_ were generated by site directed mutagenesis. DNA-sequences were assembled from pairs of designed oligonucleotides (MWG Eurofins, Ebersberg, Germany) that spanned the first 65 bases (eight forward primers for unmodified ADAPT_ERBB2-1_ and the S3A, D6A, T7A, Y10A, H11A, R14A and V15A substitutions) or last 93 bases (five reverse primers for unmodified ADAPT_ERBB2-1_ and the R35A, Y38A, A39V and A43V substitutions) of the encoding DNA with a 20 base pair overlapping section. For each variant, two oligonucleotides (100 pmol each) were assembled and extended by six cycles of PCR. The products were amplified in 35 cycles using external primers that also introduced appropriate restriction sites. For the variants S3A and A43V, clone-specific external primers were used because substitutions were located close to the ends. Purified, cleaved fragments were ligated in the expression vector.

ADAPT_ERBB2-1_, scaffold ABD and the output from the eight phage display selection tracks (A–H) were cloned into the staphylococcal display vector pSCABD1 [16]. A flexible leader sequence that is also present in the expression vector was inserted at the N-terminus of the ADAPT-variants (DEAVDANS-ADAPT). At least 60000 phagemid containing RR1ΔM15 cells were grown over night (ON) for plasmid preparation. ADAPT-inserts were amplified by PCR using Taq DNA-polymerase (produced in-house), restricted, purified and ligated into pSCABD1. The sub-libraries were transformed to electrocompetent SS320 *E. coli* (Lucigen, Middleton, WI, USA) and plasmids were prepared from ON cultures using a Jetstar 2.0 plasmid maxiprep kit (Genomed, Bad Oeynhausen, Germany) and transformed to *S. carnosus* as described previously [16].

Variants of ADAPT_ERBB2-FACS-12_ were cloned to the expression- or cell-display vector, respectively, using a 179-mer degenerate oligonucleotide (Integrated DNA Technologies, Coralville, IA, USA) that encoded all variants as a template. Degenerate codons were selected to include all possible combinations of A/V in position 28, G/D in position 33 and L/R in position 37. All variants were designed to restore the A8V substitution that was observed in the original ADAPT_ERBB2-FACS-12_ sequence. For cell binding experiments a C-terminal cysteine residue was incorporated by PCR to enable site-specific biotinylation using a biotin maleimide (Thermo Scientific, Rockford, IL, USA).

### Maturation library construction

Two different libraries were produced. In library 1 the four residues T7, Y10, H11 and Y38 were maintained whereas the remaining seven positions were randomized using NNK-codons. The ADAPT-encoding gene was assembled from two degenerate oligonucleotides (Integrated DNA Technologies) using a similar methodology as when constructing the point-mutated variants. A forward oligonucleotide with T7, Y10 and H11 conserved and NNK in the remaining four positions was assembled with a reverse oligonucleotide that retained Y38 and introduced NNK at the three remaining degenerate positions. For library 2 eight forward and four reverse oligonucleotides were mixed to partly preserve the four positions mentioned above, while still allowing some variation. A39 was also maintained to the same degree due to a lack of data on the A39V-variant. The forward oligonucleotides for library 2 either conserved all three of T7, Y10 and H11, all possible pairs of two of them, one of them or used NNK for all three positions (eight primers in total). NNK was used in the remaining 4–7 positions. The reverse oligonucleotides for library 2 contained NNK in all four randomized positions, NNK in three with Y38 or A39 conserved or NNK in two positions with both Y38 and A39 conserved (four primers in total). For library 1 150 pmol of each primer was used and for library 2 200 pmol of forward primers (mixed at an equal ratio) and 200 pmol of reverse primers (mixed at an equal ratio) were used. The assemblies were extended by six cycles of PCR and amplified in 15 cycles using Phusion DNA polymerase (NEB) and external primers that introduced restriction sites for cloning in the phagemid vector pMLII [15]. Ligation products were purified using QIAquick gel extraction kit columns and transformed into electrocompetent SS320 *E. coli* cells (Lucigen).

### Phage display selection and affinity maturation

Phage display was used to select binders to the extracellular domain of human ERBB2 from a previously described combinatorial library [15] using previously described procedures [16]. Three rounds of selection were performed using a recombinant human ERBB2-Fc chimera (R&D Systems, Minneapolis, MN, USA) as target. Phages were pre-incubated with 150 nM polyclonal human Fc (Bethyl Laboratories, Montgomery, TX, USA) for 30 min at room temperature (RT) (in rounds 2–3, not in round 1). 0.6 mg of washed and blocked Dynabeads protein A magnetic beads (Invitrogen, Carlsbad, CA, USA) were added to capture Fc and non-specifically binding phages. After pre-selection, ERBB2-Fc selection was performed using 150 nM Fc-ERBB2 for 2 h at RT in the first round before the output was split into four parallel tracks. Phages were captured using Dynabeads protein A or protein G in alternating rounds and eluted with 50 mM glycine-HCl pH 2.2.

To enrich higher affinity ERBB2-binding variants from the two affinity maturation libraries they were subjected to four rounds of phage display selection in four parallel tracks that branched out into eight tracks from cycle two (A–H). Tracks A–D derive from library 1 and tracks E–H from library 2. For each library half of the selection tracks from cycle two were performed in the presence of 1 µM unlabeled HSA (B, D, F and H) and half (A, C, E and G) were performed without HSA. ERBB2-Fc chimera (R&D Systems) was used as target in half of the tracks (C, D, G and H) and biotinylated (using Biotin-XX-succinimidyl ester B-1606, Invitrogen) ERBB2-His_6_ (Sino Biological) was used in the remaining tracks (A, B, E and F). To increase the stringency and limit avidity effects, only the monovalent ERBB2-His_6_ was used in the last cycle of all tracks. Dynabeads M280 streptavidin were used for capture in tracks with biotinylated ERBB2-His_6_ and Dynabeads Protein A for ERBB2-Fc. The number of washes was 3, 5, 10 and 15 in cycles 1-4, respectively.

### Protein expression, purification and characterization

Protein production and HSA affinity chromatography was performed essentially as described in Nilvebrant et al. [17]. IMAC was used for purification of variants that could not be recovered using HSA affinity chromatography. The pellets were resuspended in wash buffer (50 mM sodium phosphate, 300 mM NaCl and 15 mM imidazole, pH 7.5) and lysed by sonication as described before [17]. Columns containing 5 ml HisPure matrix (Thermo Scientific) were equilibrated with 10 CV of wash buffer and filtered lysates were loaded. After washing with 10 CV of wash buffer the proteins were eluted in 1 ml fractions with elution buffer (identical to the wash buffer but with 150 mM imidazole). Pure fractions were pooled and buffer exchanged to PBS using PD10 columns according to the manufacturer's recommendations (GE Healthcare, Uppsala, Sweden).

Protein molecular weights were determined by mass spectrometry on a 6520 Accurate Q-TOF LC/MS (Agilent, Santa Clara, CA, USA). Secondary structure content and melting temperatures (T_m_) were evaluated at 0.4 mg ml^−1^ by circular dichroism at 25°C using a Jasco J-810 Spectropolarimeter (Jasco, Essex, UK) as described before [17]. Melting curves were analyzed with Graphpad Prism software 5.0 by fitting a Boltzmann sigmoidal equation.

The concentration of ADAPT_ERBB2-1_ was determined by amino acid analysis (Aminosyraanalyscentralen, Uppsala, Sweden). All other protein concentrations were determined by the bicinchoninic acid (BCA) assay, using ADAPT_ERBB2-1_ as a calibrant, according to the manufacturer's protocol (Thermo Scientific).

### Surface plasmon resonance spectroscopy

SPR was used to analyze target-binding ability of the selected ADAPTs. ERBB2-Fc chimera (R&D Systems), ERBB2-His_6_ (Sino Biological, Beijing, China), polyclonal human Fc (Bethyl Laboratories) and HSA were immobilized on General Layer Medium (GLM) sensor chips using a ProteOn XPR36 Protein interaction array system (Bio-Rad, Hercules, CA, USA) essentially as described before [17]. Immobilization levels of 2100–3500 response units (RU) for ERBB2-Fc, 2000–4300 RU ERBB2-His_6_, 600 RU Fc and 1100–1600 RU HSA were used. PBST pH 7.4 was used as running buffer and 20 mM HCl for regeneration. All kinetic parameters were determined at least in duplicate using at least two different immobilization levels, dilution series or sensor chips. Data were fitted to a 1:1 Langmuir binding isotherm using the ProteOn Manager software 3.1.0.6. For alanine-variants of ADAPT_ERBB2-1_ concentrations up to 1500 nM were used in the SPR-analysis in cases when 100 nM did not result in detectable ERBB2-binding. ADAPTs with decreased albumin binding were injected at concentrations up to 5 µM. In one experiment with ten affinity-matured variants selected after the last two rounds of cell sorting, mERBB2 (Sino Biological) and mouse serum albumin (Sigma-Aldrich, St Louis, MO, USA) were also included in the analysis, they were immobilized as described above for ERBB2-His_6_ and HSA, respectively.

Potential simultaneous binding to HSA and ERBB2 was assessed by SPR using the co-inject command with injection of 100 nM of the ADAPT variant followed by either 100 nM HSA or 100 nM ERBB2-His_6_ over surfaces immobilized with ERBB2-His_6_ or HSA. As a complementary assay, 100 nM ADAPT was pre-incubated with a 5-fold molar excess of HSA for 1 h prior to injection over immobilized ERBB2-His_6_ and compared to a simultaneous injection of the ADAPT alone. In the same experiment, immobilized ADAPT_ERBB2-mat1_ was evaluated for binding to ERBB2 alone (20 nM) or 20 nM ERBB2 pre-incubated (1 h) with 100 nM of ADAPT_ERBB2-mat1_-ADAPT_ERBB2-mat10_ to verify that all candidates recognized the same epitope on ERBB2.

### Bacterial-display evaluation of ADAPT_ERBB2-1_ and pools enriched by phage display

Recombinant display on *Staphylococcus carnosus* TM300 [20] was performed as previously described [16]. The ADAPTs were subcloned to a bacterial display vector [16] in fusion to a reporter tag with affinity for human IgG. 100 nM HSA-Alexa Fluor 488-conjugate (Invitrogen, labeled according to the supplier's recommendations) or 50 nM biotinylated human ERBB2-His_6_ (Sino Biological) was used together with polyclonal human IgG-Alexa Fluor 647 conjugate (Invitrogen). The fluorescently labeled human IgG was used for monitoring the surface expression level of individual bacteria through binding to the reporter tag. The monitoring allowed normalization of the ERBB2-binding signals to minimize potential biases from differences in surface expression levels between bacteria as described previously [16]. Streptavidin-R-phycoerythrin (SA-PE) (Invitrogen) was used for detection of ERBB2. Cells were analyzed on a Gallios flow cytometer (Beckman Coulter, Brea, CA, USA). Staphylococci expressing the scaffold albumin-binding domain were used as a negative control and cells expressing ADAPT_ERBB2-1_ or an ERBB2-binding Affibody molecule (Z02891; [21]) as positive controls.

### Fluorescence-activated cell sorting

FACS was performed using an Astrios flow cytometer (Beckman Coulter). At least a 10-fold excess of cells compared to the number of *S. carnosus* transformants for track A or B from the phage display affinity maturation were washed in PBS supplemented with Pluronic and subsequently incubated with biotinylated HSA (1 µM) for 1 h at room temperature. After washing, the cells were labeled with SA-PE and IgG-Alexa Fluor 647 as described above and sorted based on their albumin-binding signal. Sorted cells were spotted directly on agar plates supplemented with 20 µg ml^-1^ chloramphenicol and their inserts were sequenced as described above.

Using a similar procedure, FACS was used to enrich the strongest ERBB2-binders from the pools derived from the eight tracks of phage display selection for affinity maturation. Cells were grown ON and the same number of cells for each track was pooled based on library design and selection strategy to form four sub-pools (tracks A+C, B+D, E+G and F+H). The cells, at least a 10 times excess to the eluted phages in the last round of phage display, were incubated with 50 nM ERBB2 (or 50 nM ERBB2 + 1 µM of HSA), labeled as described above. Fluorescent IgG was included to allow for normalization of the binding signals with the surface expression levels and the 0.2–0.8% cells with the highest ERBB2-binding signal to surface expression level ratio were collected in TSB+Y and grown ON. A second sorting round was performed using 10 nM ERBB2 (with or without 1 µM of HSA) and sorted cells were sequenced. Cells from the second round of selection were pooled into two sub-libraries (A+C+E+G and B+D+F+H) based only on selection strategy (with or without albumin) and subjected to a third round of sorting with 1 nM ERBB2 (with or without 1 µM of HSA). Incubation with 50 nM ERBB2 for 1 h followed by a wash and incubation with 100 nM of unlabelled ERBB2 for 3 h was done in the fourth round, with or without 1 µM of HSA present in all incubations with ERBB2. The top 0.3–0.7% of the cells was gated in the last two rounds and sorted cells were spread on agar plates and sequenced.

### Flow-cytometric screening

The unique clones identified after rounds three and four of FACS were screened for ERBB2-binding, HSA-binding or ERBB2-binding in the presence of unlabeled HSA using the same procedures as described above. For each variant, approximately 10^6^ cells were washed and incubated with 5 nM ERBB2, 20 nM HSA or 5 nM ERBB2 + 1 µM of unlabeled HSA. For each sample 50000 cells were analyzed for ERBB2- or HSA-binding in the flow cytometer (Gallios Beckman Coulter), all analyses were repeated in duplicate on different days and the data were normalized based on signals from scaffold ABD or ADAPT_ERBB2-1_.

### Flow-cytometric competition experiments

A competition assay was performed to evaluate if ERBB2-binding ADAPTs displayed on cells or present in solution competed with an ERBB2-specific Affibody molecule (Z02891) or an scFv derived from trastuzumab for binding to soluble ERBB2. Staphylococci expressing ADAPT_ERBB2-FACS-4_, ADAPT_ERBB2-FACS-16_, Z02891 or the trastuzumab scFv were incubated with 5 nM biotinylated ERBB2 (Sino Biological) or 5 nM biotinylated ERBB2 pre-incubated (1 h, RT) with 50 nM of each of the same proteins in solution (Z0477 [4], an anti-ERBB2 Affibody molecule that is closely related to, and recognizes the same epitope as, Z02891, was used instead of soluble Z02891). Cells were washed and incubated with secondary reagents as described above and analyzed by flow cytometry. All experiments were performed in duplicate on different days.

Staphylococcal cells expressing ADAPT_ERBB2-FACS-12 (A8)_ or variants of this molecule containing the scaffold substitutions A28V, G33D or L37R were incubated with 10 nM biotinylated ERBB2 in the presence of increasing concentrations of unlabeled HSA (0–600 µM) to assess differences in ERBB2-binding. Following washing and incubation with SA-PE as described above, ERBB2-binding was analyzed by flow cytometry. All measurements were performed in duplicate on different days. The same experiment was done using four controls: scaffold ABD, an ERBB3-specific ADAPT (ABD_ERBB3-3_, [17]) and ERBB2- (Z02891, [21]) or ERBB3-specific (Z05417, [22]) Affibody molecules.

### Binding to SKOV3-cells

SKOV-3 cells (American Tissue and Culture Collection, ATCC, Manassas, VA, USA) were grown in McCoy's 5A modified medium (Sigma Aldrich) supplemented with 10% fetal bovine serum (FBS, Sigma Aldrich) at 37°C with 5% CO_2_. Cells were harvested at 80–90% confluence by incubation with PBS supplemented with 5 mM EDTA and 0.1 mg ml^−1^ trypsin (Sigma Aldrich) for 5 min at 37°C. Between 3·10^5^–5·10^5^ cells were washed with PBS supplemented with 10% FBS and 0.1% Tween 20 and then incubated end over end with 100 nM biotinylated ADAPT at RT. For blocked samples, a 30 min pre-incubation with either 2 µM non-labeled ADAPT_ERBB2-FACS-12__(A8)_ or trastuzumab scFv was performed before the labeled ADAPT was added. Bound protein was subsequently detected with 1.25 µg ml^−1^ SA-PE by incubation on ice for 15 min and then analyzed using a Gallios flow cytometer (Beckman Coulter). All incubation reagents were diluted in PBS supplemented with 10% FBS and 0.1% Tween 20. Cells were kept on ice and centrifuged at 4°C during the washing steps to minimize dissociation of bound protein.

## Results

### Phage display selection of first generation ERBB2-binding molecules

**Figure 1:**
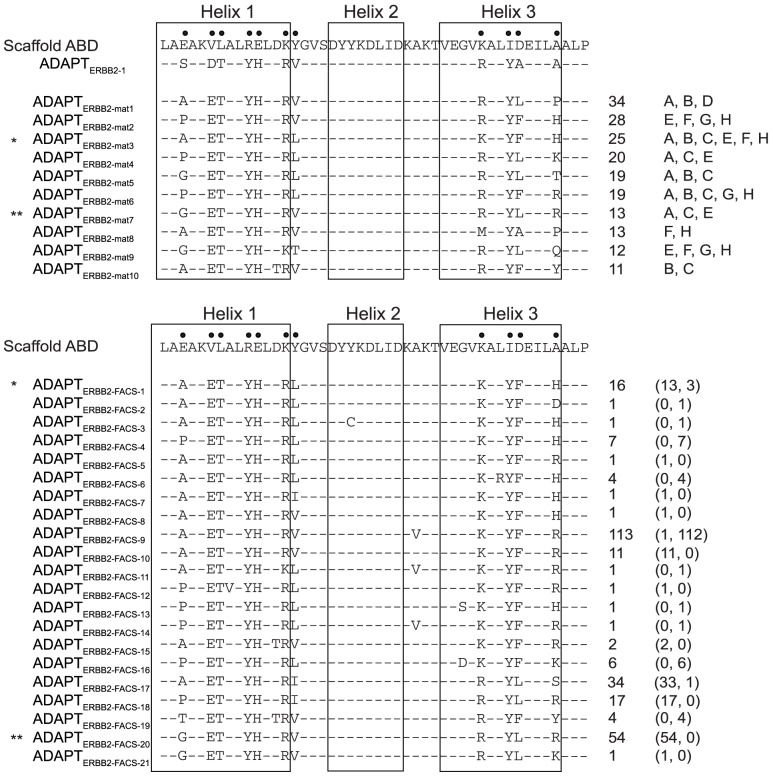
Sequences of ERBB2-binding ADAPTs. Top: Sequence of ADAPT_ERBB2-1_ and ten representative candidates found after phage display affinity maturation compared to ABD before randomization. One sequence (ADAPT_ERBB2-mat10_) contains a substitution of a scaffold residue that was not intentionally diversified in the library. The frequency of each clone in the sequence data (among 438 sequences) and tracks (A–H) where the corresponding sequence was found are indicated to the right. Track D was underrepresented in the sequence data set. Bottom: Sequences of 21 clones identified after rounds three and four of FACS sorting. The total frequency in the sequence data (278 sequences) is shown and the two numbers in brackets indicate the number of times each sequence was observed in selections without or with HSA present, respectively. Nine of the variants contained scaffold substitutions and modifications in the region spanning helix two and three were common in sequences observed after selections in the presence of HSA. 13 of these candidates were also observed after two rounds of FACS (not shown) and two of them had been observed already among the clones obtained after four rounds of phage display (identical sequences are indicated by asterisks).

Phage display selection of ADAPTs was performed in three rounds using recombinant human ERBB2 as target. DNA sequencing revealed a clear dominance of one sequence, referred to as ADAPT_ERBB2-1_ (Figure 1, Figure S1). This sequence was found in 79% of a total of 1169 colonies sequenced after the last round of selection and was found in all selection tracks. Sequencing of 180 colonies after the second round resulted in a more diverse data set, although 20% of the colonies still contained ADAPT_ERBB2-1_, which indicated that convergence on this sequence occurred early.

### Characterization of first generation candidates

The six most frequently occurring sequences were sub-cloned to an expression vector, expressed and purified. Molecular masses were verified by mass spectrometry (MS). Circular dichroism (CD) spectroscopy demonstrated that all variants shared a spectrum similar to what has been measured previously for ABD, indicating a retained three-helical structure [23]. Spectra before and after thermal denaturation could be overlapped for all proteins and melting temperatures were above 50°C for all variants. ADAPT_ERBB2-1_ denatured at 57°C (Table 1) and the parental ABD had a T_m_ above 80°C, which is comparable to what has been reported before [23].

**Table 1:** Affinities and melting temperatures (T_m_) for the non-randomized albumin-binding domain (ABD), ADAPT_ERBB2-1_ and variants of ADAPT_ERBB2-1_.

	ERBB2			HSA			
	k_a_ (M^−1^s^−1^)	k_d_ (s^−1^)	K_D_ (nM)	k_a_ (M^−1^s^−1^)	k_d_ (s^−1^)	K_D_ (nM)	T_m_ (°C)
ABD	No detectable binding			5.4 (±2.1)·10^4^	4.4 (±0.4)·10^−4^	8.1 (6)	>80
ADAPT_ERBB2-1_	2.4 (±0.4)·10^5^	1.8 (±0.3)·10^−2^	75 (n = 13)	2.3 (±1.5)·10^5^	2.4 (±0.9)·10^−4^	1.0 (8)	57
ADAPT_ERBB2-1 (S3A)_	3.4 (±0.8)·10^5^	1.4 (±0.3)·10^−2^	41 (7)	2.1 (±0.8)·10^5^	2.7 (±0.6)·10^−4^	1.3 (6)	64
ADAPT_ERBB2-1 (D6A)_	2.9 (±1.0)·10^5^	4.3 (±1.4)·10^−2^	148 (8)	1.9 (±0.9)·10^5^	3.3 (±0.6)·10^−4^	1.7 (6)	72
ADAPT_ERBB2-1 (T7A)_	No detectable binding			2.0 (±0.2)·10^5^	3.4 (±0.7)·10^−4^	1.7 (6)	63
ADAPT_ERBB2-1 (Y10A)_	No detectable binding			2.8 (±0.1)·10^5^	3.7 (±0.4)·10^−4^	1.3 (6)	61
ADAPT_ERBB2-1 (H11A)_	No detectable binding			1.1 (±0.2)·10^5^	3.5 (±0.6)·10^−4^	3.2 (6)	57
ADAPT_ERBB2-1 (R14A)_	2.5 (±0.3)·10^5^	3.5 (±0.7)·10^−2^	140 (5)	2.0 (±0.8)·10^5^	3.3 (±0.8)·10^−4^	1.7 (6)	58
ADAPT_ERBB2-1 (V15A)_	2.3 (±0.3)·10^5^	1.7 (±0.3)·10^−2^	74 (6)	3.1 (±1.9)·10^5^	3.0 (±0.3)·10^−4^	1.0 (6)	59
ADAPT_ERBB2-1 (R35A)_	3.3 (±1.2)·10^5^	4.5 (±1.3)·10^−2^	136 (5)	1.5 (±0.2)·10^5^	3.4 (±0.4)·10^−4^	2.3 (6)	55
ADAPT_ERBB2-1 (Y38A)_	No detectable binding			1.6 (±0.5)·10^5^	3.7 (±0.7)·10^−4^	2.3 (6)	52
ADAPT_ERBB2-1 (A43V)_	2.4 (±0.6)·10^5^	1.1 (±0.2)·10^−2^	46 (3)	1.4 (±0.7)·10^5^	1.9 (±1.0)·10^−4^	1.4 (6)	51

Kinetic parameters are presented as mean values with standard deviation. K_D_ was calculated from the rate constants (k_d_/k_a_) and the number of replicates for each variant is indicated within brackets. No binding to ERBB2 could be observed, even at elevated concentrations, for the negative control ABD or the four variants T7A, Y10A, H11A and Y38A. Melting temperatures (T_m_) from CD measurements are also included.

Surface plasmon resonance (SPR) spectroscopy confirmed that all variants had retained their binding to human serum albumin (HSA). However, only the most commonly observed variant, ADAPT_ERBB2-1_ (Figure 1), was able to bind ERBB2. The equilibrium dissociation constant (K_D_) for ADAPT_ERBB2-1_ was determined to 75 nM by fitting the ERBB2-binding data to a one-site binding equation (Figure S2A). The kinetic parameters are summarized in Table 1.

### Characterization of the ERBB2-binding surface of ADAPT_ERBB2-1_

Alanine mutagenesis was conducted to generate more information on the contribution of individual residues of ADAPT_ERBB2-1_ in binding to ERBB2. Variants where the eleven positions targeted for mutagenesis in the initial library were individually mutated to alanine were produced. In positions A39 and A43, the alanine residues were mutated to valine. All eleven variants were sub-cloned to an expression vector and ten of these were efficiently expressed and purified. Expression of ADAPT_ERBB2-1 (A39V)_ did not result in any detectable protein and this variant was therefore excluded from further analyses.

CD spectroscopy showed that all variants had an average helical content that was comparable to ADAPT_ERBB2-1_. The thermal melting points were shown to be between 51–72°C (Table 1). SPR was used to determine the effect of the substitutions on the affinities for ERBB2 and HSA. Four of the substitutions (T7A, Y10A, H11A and Y38A) did not yield any signal from the ERBB2-immobilized surface even at the highest concentrations, which suggested that these residues were critical for binding to ERBB2. The remaining variants all bound ERBB2 with similar kinetics as ADAPT_ERBB2-1_ (Table 1). No significant effects on albumin binding were observed for any of the substitutions.

### Design and sequence evaluation of affinity maturation libraries

The results from the alanine scan were used to design two new libraries for affinity maturation (Table 2, Figure S1). In the first library (referred to as library 1), the four residues that were shown to be crucial for ERBB2-binding were maintained while the remaining seven residues were randomized using NNK codons. In the second library (library 2), T7, Y10, H11 and Y38 were conserved to 50% and the remaining 50% was randomized by NNK. The rationale for the second design was to allow some diversity at these positions to include variants that were not present in library 1, while still favoring the residues observed in ADAPT_ERBB2-1_. Since the results from the alanine scan indicated that position 39 could be important for expression, this alanine residue was also conserved to 50% in library 2. The remaining six positions were randomized by NNK. After cloning, the phagemid libraries were transformed to *Escherichia coli* and sequence evaluation demonstrated a strong agreement with the designs.

**Table 2:** Design of affinity maturation libraries.

Position	3	6	7	10	11	14	15	35	38	39	43
ADAPT_ERBB2-1_	S	D	T	Y	H	R	V	R	Y	A	A
Library 1	*NNK*	*NNK*	T	Y	H	*NNK*	*NNK*	*NNK*	Y	*NNK*	*NNK*
Library 2	*NNK*	*NNK*	50% T	50% Y	50% H	*NNK*	*NNK*	*NNK*	50% Y	50% A	*NNK*
			50% *NNK*	50% *NNK*	50% *NNK*				50% *NNK*	50% *NNK*	

Two affinity maturation libraries were designed based on binding data of variants of ADAPT_ERBB2-1_, which showed that T7, Y10, H11 and Y38 were important for ERBB2-binding. These four residues were preserved in a conservative design (library 1) that only diversified the remaining seven positions using the degenerate codon NNK. A semi-conservative design (library 2) included 50% of conservation at the four positions that were preserved in library 1 as well as at position 39; the remaining 50% was diversified using NNK.

### Affinity maturation by phage display

The two libraries were subjected to four rounds of phage display selection against ERBB2 (Figure S1). Each library was selected in parallel against two different versions of the target protein; biotinylated His_6_-tagged ERBB2 and Fc-fused ERBB2. Two of the selection tracks were performed in an excess of unlabeled HSA in order to promote binding to ERBB2 in the presence of HSA. This resulted in eight parallel selections, called A-H. Sequencing was performed to assess the degree of convergence and a total of 438 full length ADAPT-sequences were analyzed. Logotypes that illustrate the sequence data for variants identified in selections with or without HSA are shown in figure S3.

Interestingly, the four residues that were retained in library 1 (T7, Y10, H11 and Y38) were observed in all of the 438 clones from both libraries. No differences were observed between the two forms of ERBB2 that were used as targets, which indicated that both forms were capable of presenting the epitope recognized by ADAPT_ERBB2-1_. Alanine was common in position 39 but not the most common residue whereas valine was only observed once in this position in the 438 sequenced clones. Sequence analysis also revealed a few substitutions in the scaffold regions, i.e. in positions that were not intentionally diversified in the libraries. This was mainly observed in the output from selections in the presence of HSA.

Ten clones that were frequently observed in the sequence data and also represented different regions of the global sequence cluster, different selection tracks as well as different selection pressures were chosen for characterization (ADAPT_ERBB2-mat1_ - ADAPT_ERBB2-mat10_; Figure 1). Kinetic evaluation by SPR (Table S1) revealed that all candidates bound ERBB2 with high affinities (K_D_ 0.9–9 nM) that corresponded to 8- to 83-fold improvements in K_D_ compared to ADAPT_ERBB2-1_. Albumin-binding affinities were still in the low nanomolar range. CD-measurements gave spectra similar to the scaffold ABD and melting temperatures were shown to be from 54°C to 79°C (Table S1). Furthermore, SPR was used to assess binding of ERBB2 in the presence of HSA. The assays were performed either as co-inject of the ADAPT followed by HSA over immobilized ERBB2 or by pre-incubating the ADAPT and HSA prior to injection. Although all candidates were bispecific for both ERBB2 and HSA, the analysis showed that none of the ADAPTs were capable of simultaneously binding both ERBB2 and HSA (Figure S2C).

### Cell-display of pools pre-enriched by phage display

To allow for a more comprehensive comparison of the results from the parallel tracks during phage display affinity maturation, the eight repertoires were expressed on the surface of staphylococci using an in-house developed display technology [24] and analyzed using flow cytometry. Taq polymerase was used for amplification of the phagemid inserts to allow a small degree of PCR-derived mutagenesis. Electroporation resulted in 1.4·10^5^–2.6·10^6^ transformants, hence exceeding the number of phages eluted in the last round of phage display for each track.

**Figure 2:**
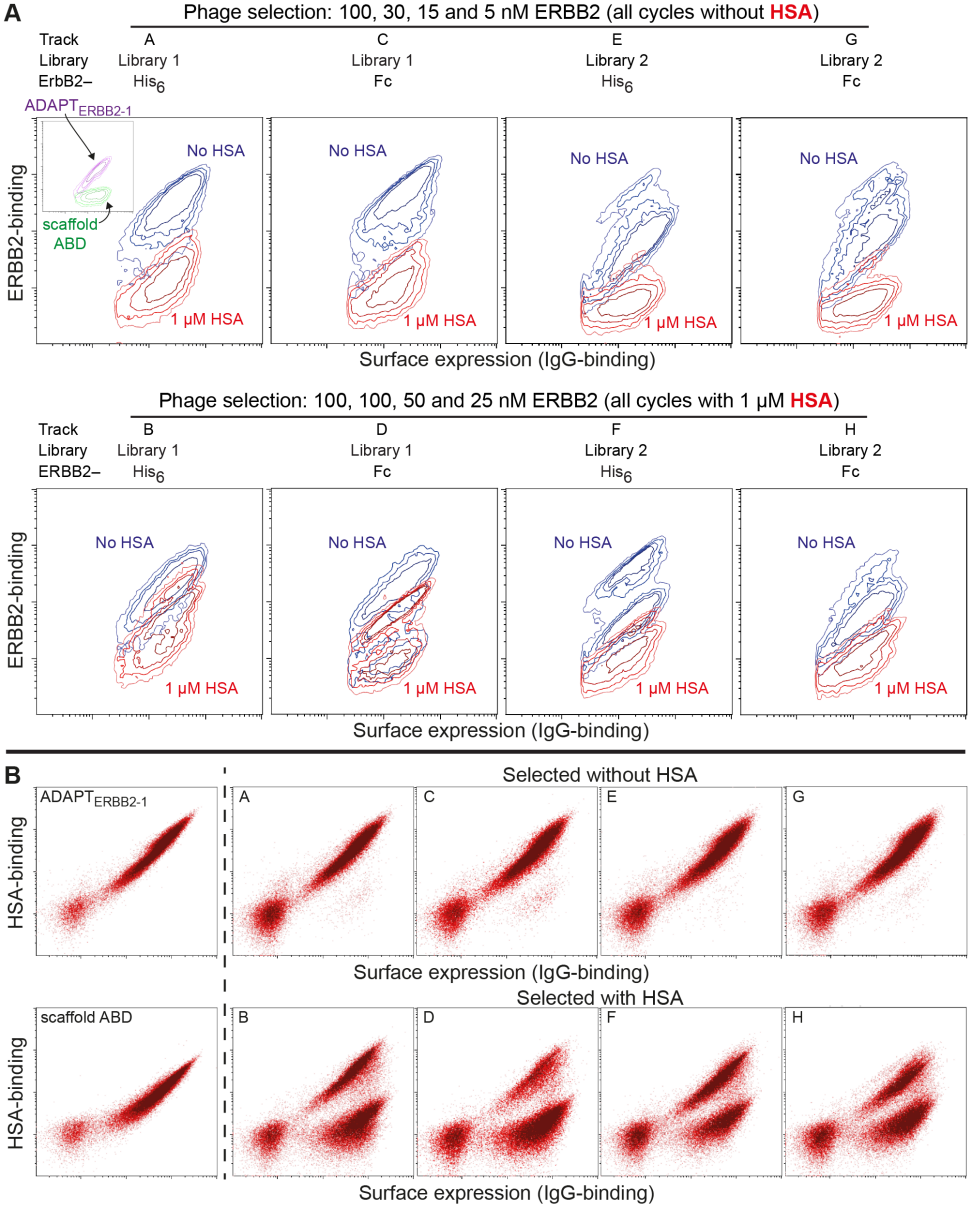
Bacterial-display analysis of batch output from selection tracks A–H. (**A**). ERBB2-binding with and without an excess of HSA. Cells expressing variants from tracks selected without (A, C, E and G) or with (B, D, F and H) 1 µM HSA present were incubated with 50 nM ERBB2 +/− 1 µM HSA and analyzed by flow-cytometry. ERBB2-binding was detected through incubation with streptavidin-R-phycoerythrin (y-axis) and surface expression levels by IgG-Alexa Fluor 647 conjugate (x-axis), data are shown using logarithmic scales. Each diagram presents an overlay of contour plots of the expressing populations of cells from two separate samples from the same batch (A-H) incubated with only ERBB2 (blue) or ERBB2 and HSA (red). The contours represent cell density in the respective regions corresponding to 20, 40, 60 or 80% of the maximum cell density observed. Each sample is represented by 25–30000 events. Corresponding data for ADAPT_ERBB2-1_ and scaffold ABD are shown in the inlay of panel A to facilitate a comparison. The library each track is derived from, the form of ERBB2 and concentrations used during phage display selection are also indicated. **(B**). HSA-binding. Cells from tracks selected without (A, C, E and G) or with (B, D, F and H) 1 µM HSA present were incubated with 50 nM HSA-Alexa Fluor 488 conjugate and analyzed by flow-cytometry. HSA-binding is shown on the y-axis and surface expression level, detected by IgG-Alexa Fluor 647 conjugate, on the x-axis. As controls, cells expressing ADAPT_ERBB2-1_ or scaffold ABD are included.

As expected, a flow-cytometric analysis demonstrated enrichment of ERBB2-binding variants in all tracks (Figure 2A). However, some differences between the repertoires were revealed. The enrichment of high-affinity clones was more pronounced for library 1 (A–D) compared to library 2 (E–H). Moreover, when ERBB2-binding was analyzed in the presence of a 20-fold molar excess of unlabeled HSA, the tracks selected with HSA present (B, D, F and H) showed higher ERBB2-binding signals compared to tracks selected without HSA (A, C, E and G) (Figure 2A). To investigate this further, cells from all tracks were incubated with labeled HSA and analyzed for HSA binding in the flow cytometer. The analysis revealed that the majority of clones selected with only ERBB2 retained their binding to HSA (Figure 2B). However, clones selected in an excess of HSA (tracks B, D, F and H) formed two distinct populations with high and low binding signal to HSA, respectively. To further investigate these two clusters, the cell populations of track B (Figure 2B) were sorted in HSA-binding and non-binding populations using fluorescence-activated cell sorting (FACS) followed by DNA-sequencing (Figure S4). Strikingly, 95% of the clones in the non-binding population contained the scaffold substitution A28V or a proline residue in position 43, indicating the importance of these positions for albumin binding. None of these sequence features was observed in any of the clones derived from the population sorted for HSA-binding or a control population sorted from track A.

### Selection by fluorescence-activated cell sorting

The staphylococcal-displayed outputs from the eight phage display selections (Figure 2A) were pooled based on library design and selection strategy (with or without HSA present, Figure S4). FACS was used to enrich the cells expressing the strongest ERBB2-binders. Unlabeled HSA was added to the pools that had previously been selected in presence of high concentrations of albumin. Many unique clones were observed after two rounds of FACS, which indicated that further sorting was necessary. Sequencing of more than 270 clones after the last two rounds of FACS revealed 21 unique variants (ADAPT_ERBB2-FACS1-21_, Figure 1). Two of these variants were identical to clones identified after the second phage display selection. Interestingly, nine of the 21 variants contained substitutions in scaffold regions that were not intended in the library design. Five different substitutions were observed among clones derived from selections with HSA present (Y20C, A28V, G33S, G33D and L37R) and two were found without HSA (A8V and D13T).

### Screening and characterization of chosen variants

**Figure 3:**
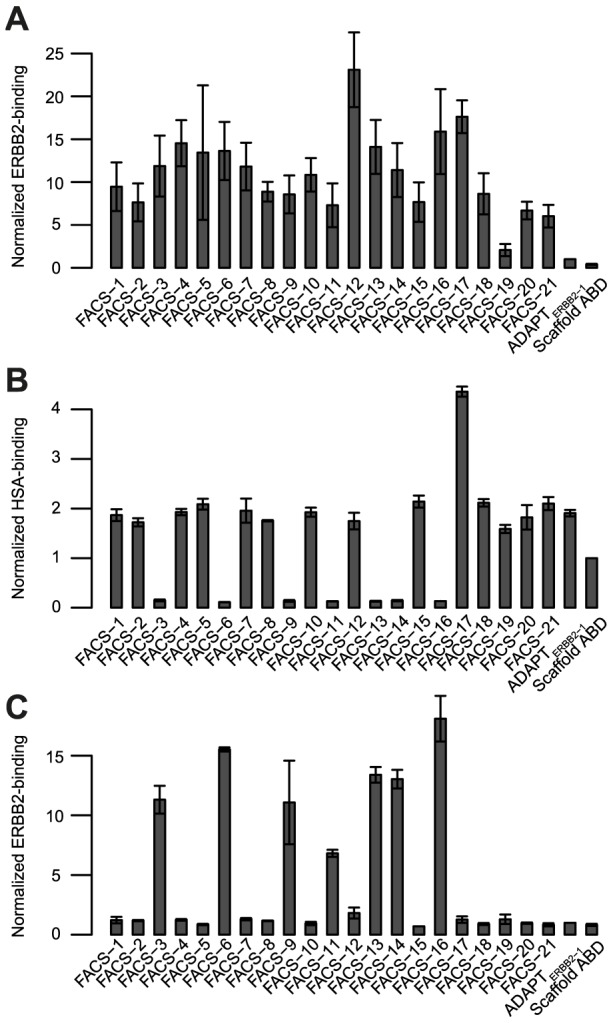
Bacterial-display screening of variants identified after FACS. 21 clones identified after rounds three and four of FACS sorting were screened for ERBB2-binding (**A**), HSA-binding (**B**) and ERBB2-binding in the presence of an excess of HSA (**C**). 5 nM ERBB2 was used in screen A and the binding signal divided by the surface expression level for each clone was normalized against the signal from ADAPT_ERBB2-1_. 20 nM HSA was used in screen B and the signals were normalized to scaffold ABD. 5 nM ERBB2 together with 1 µM unlabeled HSA was used in screen C and ADAPT_ERBB2-1_ was used for normalization. All measurements were done in duplicate on different days and the bars indicate the standard deviation.

Three flow-cytometric screens were performed with the 21 variants expressed on the surface of staphylococcal cells (Figure 3A–C). Binding to ERBB2 (Figure 3A), HSA (Figure 3B) and ERBB2 in the presence of an excess of unlabeled HSA (Figure 3C) were assessed. ADAPT_ERBB2-1_ and scaffold ABD were included as controls and used to normalize the ERBB2- and HSA-binding signals, respectively, between runs. The ERBB2-screen showed that variants with improved affinities compared to ADAPT_ERBB2-1_ had been selected. All variants bound ERBB2 and ADAPT_ERBB2-FACS-12_ gave the strongest signal. Interestingly, the HSA-binding was strongly influenced by scaffold substitutions in the region spanning helices two and three. All variants with substitutions in this region lost their strong binding to HSA whereas the variants lacking scaffold alterations or having substitutions in the first helix (A8V and D13T) still bound albumin. Moreover, only variants that lost their binding to HSA retained their ability to bind ERBB2 when the binding analysis was performed in the presence of an excess of albumin.

Based on sequences and data from the screening, ten new variants were selected for detailed characterization. ADAPT_ERBB2-FACS-4, 5, 6, 9, 10, 12, 13, 16, 17 and 18_ (Figure 1) were expressed in *E. coli* with an N-terminal His_6_-tag. Variants without scaffold substitutions in helices two to three were purified using HSA affinity chromatography and the remaining variants were purified by immobilized metal ion affinity chromatography (IMAC).

Analysis using CD-spectroscopy gave similar spectra as for scaffold ABD and melting points between 49–80°C (Table 3). ADAPT_ERBB2-FACS-12_, the strongest ERBB2-binder in the screen that also contained the substitution A8V in the first helix, did not refold fully after heating. Reverting the mutation back to alanine (V8A) restored the solubility and this variant was called ADAPT_ERBB2-FACS-12 (A8)_. Binding analysis by SPR gave strong ERBB2-affinities (K_D_) for all variants, ranging from 0.3-2.5 nM (Table 3). ADAPT_ERBB2-FACS-12_ demonstrated around 250-fold improved affinity compared to the initial variant ADAPT_ERBB2-1_ (Figure S2A–B). The albumin binding affinities were similar to previously characterized variants (0.6–1.3 nM K_D_), except for the variants with scaffold substitutions spanning the two last helices (Figure 1, Table 3). The SPR-analysis also included the murine forms of ERBB2 and albumin. No binding was observed to murine ERBB2 and analysis of binding to mouse serum albumin (MSA) gave roughly 10-fold weaker binding compared to HSA, a pattern that has been observed earlier [17].

**Table 3:** Affinities of selected affinity matured ADAPTs after FACS.

	ERBB2 (human)			HSA			MSA			
	k_a_ (M^−1^s^−1^)	k_d_ (s^−1^)	K_D_ (nM)	k_a_ (M^−1^s^−1^)	k_d_ (s^−1^)	K_D_ (nM)	k_a_ (M^−1^s^−1^)	k_d_ (s^−1^)	K_D_ (nM)	T_m_ (°C)
ADAPT_ERBB2-FACS-4_	1.7(±0.8)·10^5^	9.6(±3.5)·10^−5^	0.6 (n = 4)	1.1(±0.4)·10^5^	7.0(±3.5)·10^−5^	0.6 (3)	5.5(±2.0)·10^4^	2.8(±0.2)·10^−4^	5.1 (2)	62
ADAPT_ERBB2-FACS-5_	2.2(±0.4)·10^5^	3.0(±0.3)·10^−4^	1.4 (6)	1.6(±0.2)·10^5^	1.3(±0.1)·10^−4^	0.8 (6)	N.D.			80*
ADAPT_ERBB2-FACS-6_	2.7(±0.2)·10^5^	1.4(±0.1)·10^−4^	0.5 (4)	No detectable binding			No detectable binding			64
ADAPT_ERBB2-FACS-9_	2.6(±0.3)·10^5^	2.1(±1.0)·10^−4^	0.8 (4)	No detectable binding			No detectable binding			64
ADAPT_ERBB2-FACS-10_	2.6(±0.8)·10^5^	2.1(±1.1)·10^−4^	0.8 (4)	1.6(±0.7)·10^5^	1.6(±0.9)·10^−4^	1.0 (2)	6.0·10^4^	8.3·10^−4^	14 (1)	68
ADAPT_ERBB2-FACS-12_	2.1(±0.9)·10^5^	6.7(±2.9)·10^−5^	0.3 (4)	1.1(±0.7)·10^5^	1.1(±0.5)·10^−4^	1.0 (2)	2.4·10^4^	5.4·10^−4^	14 (1)	63
ADAPT_ERBB2-FACS-13_	0.5(±0.1)·10^5^	1.2(±0.4)·10^−4^	2.5 (4)	Weak binding			Weak binding			N.D.
ADAPT_ERBB2-FACS-16_	1.4(±0.5)·10^5^	9.0(±1.1)·10^−5^	0.6 (2)	No detectable binding			No detectable binding			72
ADAPT_ERBB2-FACS-17_	2.5(±0.9)·10^5^	5.7(±2.9)·10^−4^	2.2 (4)	1.1(±0.4)·10^5^	1.4(±0.9)·10^−4^	1.3 (2)	5.6·10^4^	7.9·10^−4^	14 (1)	78
ADAPT_ERBB2-FACS-18_	1.9(±0.8)·10^5^	3.2(±1.5)·10^−4^	1.7 (4)	0.9(±0.5)·10^5^	8.9(±5.2)·10^−5^	1.0 (2)	3.2·10^4^	4.6·10^−4^	14 (1)	49
ADAPT_ERBB2-FACS-12 (A8)_	1.6(±0.3)·10^5^	1.6(±0.2)·10^−4^	1.0 (6)	9.8(±0.2)·10^4^	1.4(±0.2)·10^−4^	1.4 (4)	N.D.			61
ADAPT_ERBB2-FACS-12 (A8) A28V_	4.5(±1.2)·10^4^	2.0(±0.4)·10^−4^	4.4 (6)	No detectable binding			N.D.			N.D.
ADAPT_ERBB2-FACS-12 (A8) G33D_	2.7(±0.4)·10^5^	1.7(±0.2)·10^−4^	0.6 (6)	No detectable binding			N.D.			72
ADAPT_ERBB2-FACS-12 (A8) L37R_	1.4(±0.3)·10^5^	9.7(±0.4)·10^−5^	0.7 (6)	No detectable binding			N.D.			45
ADAPT_ERBB2-FACS-12 (A8) A28V, G33D, L37R_	2.8(±1.5)·10^4^	1.6(±0.1)·10^−4^	5.8 (6)	No detectable binding			N.D.			N.D.

Kinetic parameters are presented as mean values with standard deviation. K_D_ was calculated from the rate constants (k_d_/k_a_) and the number of replicates for each variant is indicated within brackets. Ten variants were selected for kinetic analysis. Variants of the strongest binder, ADAPT_ERBB2-FACS-12_, with different substitutions were also characterized. The binding kinetics and melting temperatures for some interactions were not determined (N.D.). No binding was observed to murine ERBB2.

*Determined by manual inspection of melting curve.

### Detailed investigation of the interaction with albumin

Sequencing and affinity measurements suggested that some single amino acid substitutions decreased the affinity for albumin. To verify these indications, three substitutions (A28V, G33D and L37R) were grafted to one of the variants with retained binding to albumin and strong ERBB2-binding (ADAPT_ERBB2-FACS-12 (A8)_). Four clones were prepared, containing either one of these three selected substitutions, respectively, and a variant with all three substitutions in the same construct. Analysis confirmed that all proteins were soluble and retained a high affinity for ERBB2 (Table 3). Most variants retained the sub-nanomolar ERBB2-affinities; both variants with A28V displayed a small decrease in association rate. No binding to albumin was observed for these four variants at concentrations up to 5 µM, thus confirming the importance of these positions for binding to albumin.

**Figure 4:**
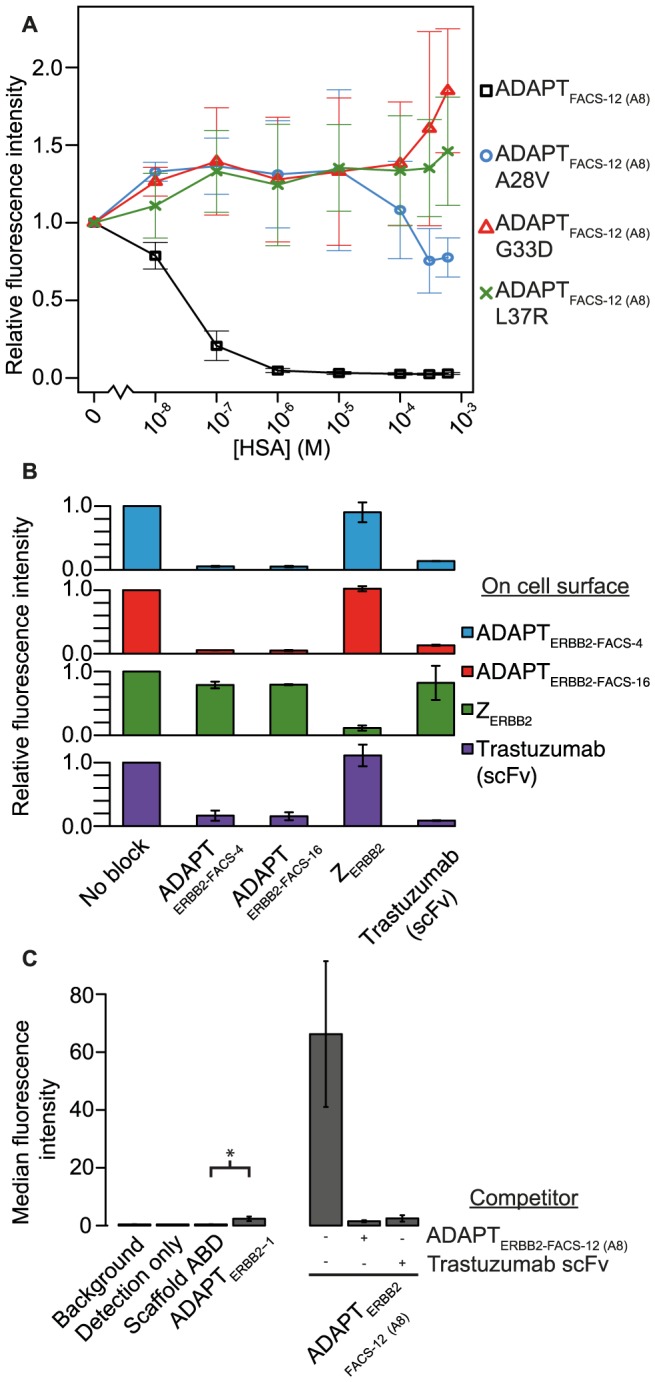
Analysis of affinity matured ERBB2-binders. (**A**). ERBB2-binding at high concentrations of HSA. Four variants of ADAPT_ERBB2-FACS-12 (A8)_ were displayed at the bacterial surface and incubated with labeled ERBB2 (10 nM) in the presence of varying concentrations of HSA. All mutated variants show a retained ability to bind ERBB2 at very high concentrations of albumin, which is in contrast to the parental ADAPT_ERBB2-FACS-12 (A8)_. Increasing streptavidin-R-phycoerythrin signals were observed at high concentrations of HSA. This effect was only seen for molecules with retained ERBB2-binding and was also observed for an ERBB2-binding Affibody molecule used as control (Figure S5). Hence, this experimental artifact was not ADAPT-specific. All measurements were done in duplicate on different days and the bars indicate the standard deviation. The albumin concentration is shown on a logarithmic scale, the two highest concentrations were 300 and 600 µM, respectively. (**B**). Competition experiment using ERBB2-binding proteins expressed on staphylococcal cells and flow cytometry. ERBB2-binding (5 nM) to cell-displayed binders was always blocked by a 10-fold excess of the same protein in a soluble form. Only ADAPTs and trastuzumab competed with each other for ERBB2-binding, no competition with the ERBB2-binding Affibody molecule Z_ERBB2_ was observed. All measurements were done in duplicate on different days and the bars indicate the standard deviation. (**C**). Binding to native ERBB2 on SKOV-3 cells. Cells were incubated with 100 nM biotinylated ADAPT_ERBB2-1_ or ADAPT_ERBB2-FACS-12__(A8)_ and detected with streptavidin-R-phycoerythrin conjugate. Pre-incubating the cells with an excess of either non-labeled ADAPT_ERBB2-FACS-12 (A8)_ or trastuzumab scFv blocked binding of ADAPT_ERBB2-FACS-12 (A8)_. A two-sample two-tailed t-test demonstrated that the binding for ADAPT_ERBB2-1_ was significantly stronger than the signal from the scaffold control (p = 0.0067). All measurements were done in duplicate on different days and the bars indicate the standard deviation. Analyses of non-treated cells were included in all experiments (background).

To further investigate the grafted substitutions, a competition assay in which staphylococcal display was used to assess ERBB2-binding at physiologically relevant albumin concentrations was performed. Cells expressing the variant ADAPT_ERBB2-FACS-12 (A8)_, or the same binder with one of the scaffold substitutions A28V, G33D, L37R, were incubated with labeled ERBB2 (10 nM) and a titration of unlabeled HSA (0–600 uM). ADAPT_ERBB2-FACS-12 (A8)_, which has an intact albumin-binding region, quickly lost its ability to bind ERBB2 when the HSA-concentration was increased (Figure 4A). In contrast, the mutants with deleted albumin binding were able to bind ERBB2 even at near physiological concentrations of HSA. An ERBB2-binding Affibody molecule was used as a control and bound ERBB2 over the whole concentration range of HSA.

### Trastuzumab competition

Because all ADAPTs were derived from the same first generation ERBB2-binding molecule, they were expected to recognize the same epitope on ERBB2. To investigate if this epitope was overlapping with the binding sites of an ERBB2-specific Affibody molecule (Z_ERBB2_) [21] or the anti-ERBB2 antibody trastuzumab (Herceptin), a flow-cytometry based blocking assay was performed. Staphylococcal cells expressing either Z_ERBB2_, an scFv derived from trastuzumab or ERBB2-binding ADAPTs were incubated with only ERBB2 or ERBB2 pre-incubated with an excess of each of the same four proteins in a soluble form. Flow cytometry was used to measure ERBB2 bound to the cells. As expected, the assay showed that all soluble binders were able to block the binding of ERBB2 to cells expressing the same molecule that was used for blocking (Figure 4B). However, only the ADAPTs and trastuzumab could cross-inhibit each other and neither the ADAPTs nor trastuzumab could compete with the Affibody molecule. These results demonstrated that the ERBB2-binding ADAPT molecules bind domain IV of ERBB2 at a site that is overlapping with the known epitope of trastuzumab [25]. The lack of competition with the Affibody molecule, which binds an epitope mainly located in domain III [26], was also consistent with this finding.

### Binding to human cancer cells

To verify that the ERBB2-binding ADAPTs could recognize native ERBB2 on human cells, labeled ADAPTs were incubated with SKOV-3 cells and analyzed using flow cytometry. Cell binding was observed for both ADAPTs and the signal correlated with the affinity (Figure 4C). To show that the observed signal was due to specific binding, a pre-incubation was made with an excess of non-labeled competitor, either ADAPT_ERBB2-FACS-12 (A8)_ itself or an scFv derived from trastuzumab. The binding signal was significantly decreased by pre-incubation with both competitors (Figure 4C).

## Discussion

The aim of this study was to generate small, bispecific binding proteins with ability to bind ERBB2 with high affinity and to evaluate the influence of the inherent HSA binding. Such molecules could potentially combine favorable properties of a very small size and a prolonged, and possibly adaptable, serum half-life. To achieve this, combinatorial protein engineering of an albumin-binding domain was performed using a combination of phage and bacterial display technologies. The scaffold chosen for this, ABD, has many attractive features, such as a high stability, high solubility, lack of disulfide bonds and a high tolerance to amino acid substitutions [27]. The initial phage display selection identified one ERBB2-binding variant (ADAPT_ERBB2-1_) with favorable biophysical properties, a high affinity for HSA and a moderate affinity for ERBB2 (Figure 1, Table 1). Based on alanine-scanning data of the ERBB2-binding surface, two affinity maturation libraries were designed (Table 2). Phage display selections from the new libraries yielded several variants with higher affinity to ERBB2 as well as variants able to bind ERBB2 in a high molar excess of HSA. Four residues (T7, Y10, H11 and Y38), also recognized as important in the alanine scanning experiment, were observed in all 438 identified clones. This suggests that they provided an important hot spot for the interaction with ERBB2.

The output from the phage display selections was transferred to a bacterial-display system to enable further analysis and affinity maturation. A comparison of ERBB2-binding with and without HSA present clearly demonstrated that selections with HSA present had enriched for molecules with ability to bind ERBB2 under high albumin concentrations (Figure 2A). Interestingly, analysis of the HSA-binding demonstrated that this was due to a strong enrichment of variants with reduced albumin binding (Figure 2B). We identified several scaffold substitutions that resulted in diminished affinity for albumin (Table 3). Hence, selection for ERBB2-binding in presence of HSA promoted decreased affinity for albumin. Binding to ERBB2 and HSA was assessed by SPR experiments and although the assay showed that a number of clones were bispecific with high affinity to both albumin and ERBB2, none were able to bind both targets simultaneously (Figure 3, Figure S2C).

In order to narrow down the diverse set of variants that was identified after phage display affinity maturation, FACS was used to enrich the staphylococcal-displayed affinity maturation libraries (Figure S4). Cell-display and FACS is attractive for maturation efforts since it enables fine affinity discrimination by application of stringent sort gates. 21 unique candidates were identified after the final rounds of cell sorting, out of which two had been detected already after phage display (Figure 1). Analysis of these 21 candidates revealed a clear connection between loss of albumin binding and substitutions in the albumin-binding region of the domains (Figure 3). Several substitutions occurred in clones with non-identical DNA-sequences, which shows that they were independently enriched in more than one individual clone. Most of these were probably introduced during amplification of the phagemid inserts prior to cloning into the cell display vector. Taq polymerase was used to intentionally introduce a low degree of additional diversity in the redundant pool of sequences derived from the phage display selections. Sequencing of clones after two or four rounds of FACS showed that this strategy was successful and that variants with scaffold substitutions were enriched during selections with albumin present. 8% of the sequenced clones contained additional substitutions after four rounds of phage display selection in the two maturation libraries. The large increase of the proportion of clones with substitutions in the following selections with albumin present (51% of the sequenced clones after two rounds of cell sorting and 92% after rounds 3–4) illustrates that the scaffold needed to adjust to the tough selection pressure. In this case the solution was to diminish the albumin binding when simultaneous binding to the target could not be achieved.

During affinity maturation, the hot spot of ADAPT_ERBB2-1_ had been supplemented with novel beneficial residues to generate variants with up to 250-fold improved affinity for ERBB2 compared to the initial binding molecule (Table 3, Figure S2). The large amount of data of the more than 20 clones that have been expressed and characterized enabled an assessment of the importance of some residues for strong affinity. For example, pair-wise comparisons of variants ADAPT_ERBB2-FACS-5_ and ADAPT_ERBB2-FACS-12_ or ADAPT_ERBB2-mat-1_ (K_D_ 2.3 nM, see Table S1) and ADAPT_ERBB2-FACS-4_ (Figure 1, Table 3) indicated that a proline residue in the first randomized position is beneficial for ERBB2-binding. Three substitutions that influenced the albumin-binding ability (A28V, G33D and L37R) were selected for more thorough characterization. Available annotations of albumin-binding residues in similar albumin-binding domains supported the significance of these positions in the native interaction between ABD and HSA [28], [29]. To facilitate direct assessments of the three selected substitutions, they were evaluated in the context of the same affinity-matured ERBB2-binder; ADAPT_ERBB2-FACS-12 (A8)._ The SPR-results showed that each substitution alone could decrease the albumin binding to an undetectable level and that all three modifications also could be combined in the same molecule. The ERBB2-binding was essentially unaffected by all mutations except A28V (Table 3). However the decreased association rate constant linked to this substitution was not observed for other variants where only position 28 differed (ADAPT_ERBB2-FACS-9_ and ADAPT_ERBB2-FACS-10_; Table 3) Cell-display with ADAPT_ERBB2-FACS-12 (A8)_ containing A28V, G33D or L37R verified that the HSA affinity dramatically affected the ability of the molecules to bind ERBB2 in high concentrations of HSA (Figure 4A).

A competition experiment using different ERBB2-binders displayed on staphylococci revealed that the ADAPTs competed with trastuzumab for binding to the receptor (Figure 4B). A well-characterized Affibody molecule that recognizes a different epitope on ERBB2 was also included in these experiments. The finding that the ADAPTs recognize a different site than the Affibody molecule is interesting considering the structural similarity between these scaffolds. Previous selections, for example targeting ERBB3 [17], have generated ADAPTs directed to the same epitope as previously selected Affibody molecules [30]. This feature may allow new fusion formats that target two distinct ERBB2-epitopes to be constructed. A similar strategy using designed ankyrin repeat proteins targeted to different epitopes on ERBB2 has shown a high potential for cancer treatment *in vitro* that did not rely on the addition of functional payloads [31]. Furthermore, binding assays on ERBB2-positive human cancer cells demonstrated that the ADAPTs specifically recognized the native receptor (Figure 4C).

Earlier studies have validated that albumin binding has a great potential for improving the properties of ERBB2-binding proteins. Dennis et al. improved the pharmacokinetics of an ERBB2-specific Fab-fragment by fusion of an albumin-binding peptide [32]. ERBB2-binding has also recently been engineered into an Fc-fragment for similar purposes [33]. However, the molecules in these examples are roughly ten times larger than the ADAPTs described in this paper. Experiments using ABD-fusions to an ERBB2-specific Affibody molecule have demonstrated a dramatic decrease in kidney accumulation, an effectively prolonged half-life and an improved tumor accumulation compared to non-fused controls [34]. It has also been shown that a similar Affibody-ABD fusion protein did not interfere with the FcRn-albumin binding kinetics [35]. Hence, ABD-fusions can use albumin as a carrier both to avoid renal filtration and to escape endosomal degradation. Consequently, a protein of small size and high affinity for the target protein in combination with albumin binding capacity, such as the bispecific ADAPTs described here, has great potential to meet clinical challenges. To achieve a bispecific ADAPT that can exploit HSA for delayed renal filtration, the relation between the two affinities has to be optimized. A low HSA-affinity in combination with a strong and specific target binding might endow the domain with target specificity as well as long half-life.

To conclude, we have used combinatorial protein engineering together with phage- and cell-display technology to design, evolve and evaluate new minimal binding proteins based on an albumin-binding domain toward the cancer-associated ERBB2. The experimental design of the selections provided the possibility to achieve binding domains with different properties. Bispecific protein domains with high affinity to both ERBB2 and albumin were attained, although no simultaneous binding could be detected. The interplay between the strength of the two different binding activities and their impact on the distribution within the body is of high interest. The results from this study identified single substitutions for regulating the albumin affinity and are hence important findings for such further investigations *in vivo*. The possibility to independently fine-tune each interaction gives us an opportunity to use the ADAPTs for different applications. ADAPTs without affinity for HSA have potential for molecular imaging applications, while variants with low affinity for HSA might be more suitable for therapeutic applications. These different areas of application will be in focus in coming studies.

## Supporting Information

Figure S1 **Overview of the phage display selections.**
(EPS)

Figure S2 **SPR analysis of ERBB2 binding ADAPTs. (A).** ADAPT_ERBB2-1_ binding to immobilized ERBB2. Response units for ERBB2-binding at different concentrations of ADAPT_ERBB2-1_ are shown on the y-axis and time (s) on the x-axis. **(B).** Affinity-matured variant ADAPT_ERBB2FACS-12_ binding to immobilized ERBB2. **(C)** Injection of affinity-matured variant ADAPT_ERBB2-mat1_ (100 nM) over immobilized ERBB2 directly followed by an injection of HSA (100 nM).
(EPS)

Figure S3 **Sequence logotypes from clones identified after phage display selections with and without HSA in the selection steps.** By DNA-sequencing 118 unique (out of 271 sequences) without HSA (35 repeated and one occurring 19 times (16%)) and 50 unique sequences (out of 167 sequences) with HSA (19 repeated and the most common 32 times (19%)) were identified The eleven randomized positions are shown from left (N-terminus) to right (C-terminus) and their locations in the 46 amino acid ABD sequence are indicated by numbers. Logotypes were generated using Weblogo 3.3 [36]. The overall height of each residue corresponds to its degree of conservation and the height within each stack relates to the relative frequency. The maximum sequence conservation per site is described by log_2_(20) for 20 possible amino acids (≈4.3 bits). Sequences obtained from both libraries are included. **(A).** Sequence logotypes from selections without HSA present. (**B).** Corresponding logotype for sequences from selections with HSA present. The main difference to A. is that proline was the most common residue in position 43 in this data set (35%; 1% in selections without HSA) and that the scaffold substitution A28V (not shown in the logotypes) was very common in sequences derived from selections with HSA present.
(TIFF)

Figure S4 **Overview of cell sortings during affinity maturation (A).** Eight selection tracks (A–H) from the two phage display libraries (library 1 and 2) where displayed on staphylococcal cells to enable fluorescence-activated cell sorting for ERBB2-binding. Cells from tracks A and B were sorted based on HSA-binding and sequenced before the first cycle of ERBB2-selection. Cells were pooled in four sub-pools based on the previously applied selection strategy (with or without 1 µM unlabelled HSA present) and library design (Library 1 or 2). All pools were selected for ERBB2-binding both with and without excess albumin present in eight sorting experiments for two rounds. After two rounds of sorting, individual clones were sequenced and the outputs from the second cycle were pooled only based on selection strategy (into two sub-pools). DNA sequencing after the last two sorting cycles identified 21 unique sequences. Sort gates and the percentage of analyzed cells that was sorted in each experiment are indicated in each diagram. ERBB2-binding (measured as fluorescence from streptavidin-R-phycoerythrin) is shown on the y-axis and surface expression level (measured as fluorescence from bound IgG conjugated to Alexa Fluor 647) is shown on the x-axis, data are shown using logarithmic scales. Surface expression level was monitored to allow normalization of the ERBB2-binding signal and minimize potential biases from differences in surface expression levels.
(TIF)

Figure S5 **Binding of ERBB2 to Z_ERBB2_ and other control proteins expressed on the surface of staphylococcal cells in the presence of unlabeled HSA**. An apparent increase in binding signal is observed at high concentrations of HSA for the ErBB2-binder. Scaffold ABD, an ERBB3-binding ADAPT (ADAPT_ERBB3-3_) and an ERBB3-binding Affibody molecule (Z_ERBB3_) were included as controls.
(EPS)

Table S1 **Affinities of selected affinity-matured ADAPTs after phage display.** Kinetic parameters are presented as mean values with standard deviation. K_D_ was calculated from k_d_/k_a_ and the number of replicates is indicated within brackets. Ten selected molecules, ADAPT_ERBB2-mat1_-ADAPT_ERBB2-mat10_, were included in the analysis.
(DOCX)
