# Supplementary figures and images for: Engineering of Bispecific Affinity Proteins with High Affinity for ERBB2 and Adaptable Binding to Albumin
Source: PLoS One. 2014 Aug 4;9(8):e103094. doi: 10.1371/journal.pone.0103094 (PMC4121139; doi:10.1371/journal.pone.0103094)

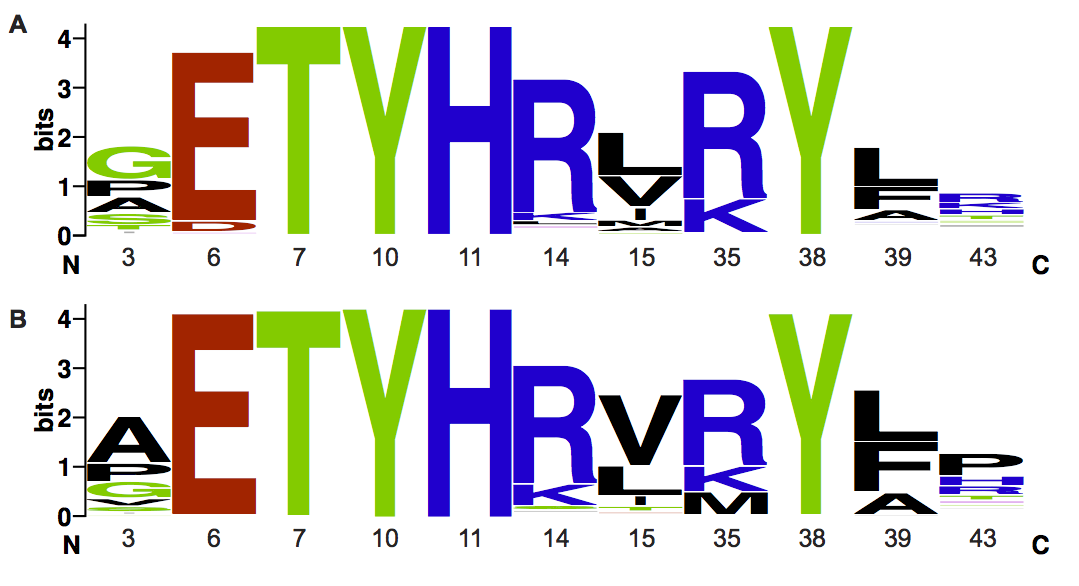

Supplement: Figure S3 — Sequence logotypes from clones identified after phage display selections with and without HSA in the selection steps. By DNA-sequencing 118 unique (out of 271 sequences) without HSA (35 repeated and one occurring 19 times (16%)) and 50 unique sequences (out of 167 sequences) with HSA (19 repeated and the most common 32 times (19%)) were identified The eleven randomized positions are shown from left (N-terminus) to right (C-terminus) and their locations in the 46 amino acid ABD sequence are indicated by numbers. Logotypes were generated using Weblogo 3.3 [36]. The overall height of each residue corresponds to its degree of conservation and the height within each stack relates to the relative frequency. The maximum sequence conservation per site is described by log2(20) for 20 possible amino acids (≈4.3 bits). Sequences obtained from both libraries are included. (A). Sequence logotypes from selections without HSA present. (B). Corresponding logotype for sequences from selections with HSA present. The main difference to A. is that proline was the most common residue in position 43 in this data set (35%; 1% in selections without HSA) and that the scaffold substitution A28V (not shown in the logotypes) was very common in sequences derived from selections with HSA present. (TIFF) [file pone.0103094.s003.tiff]

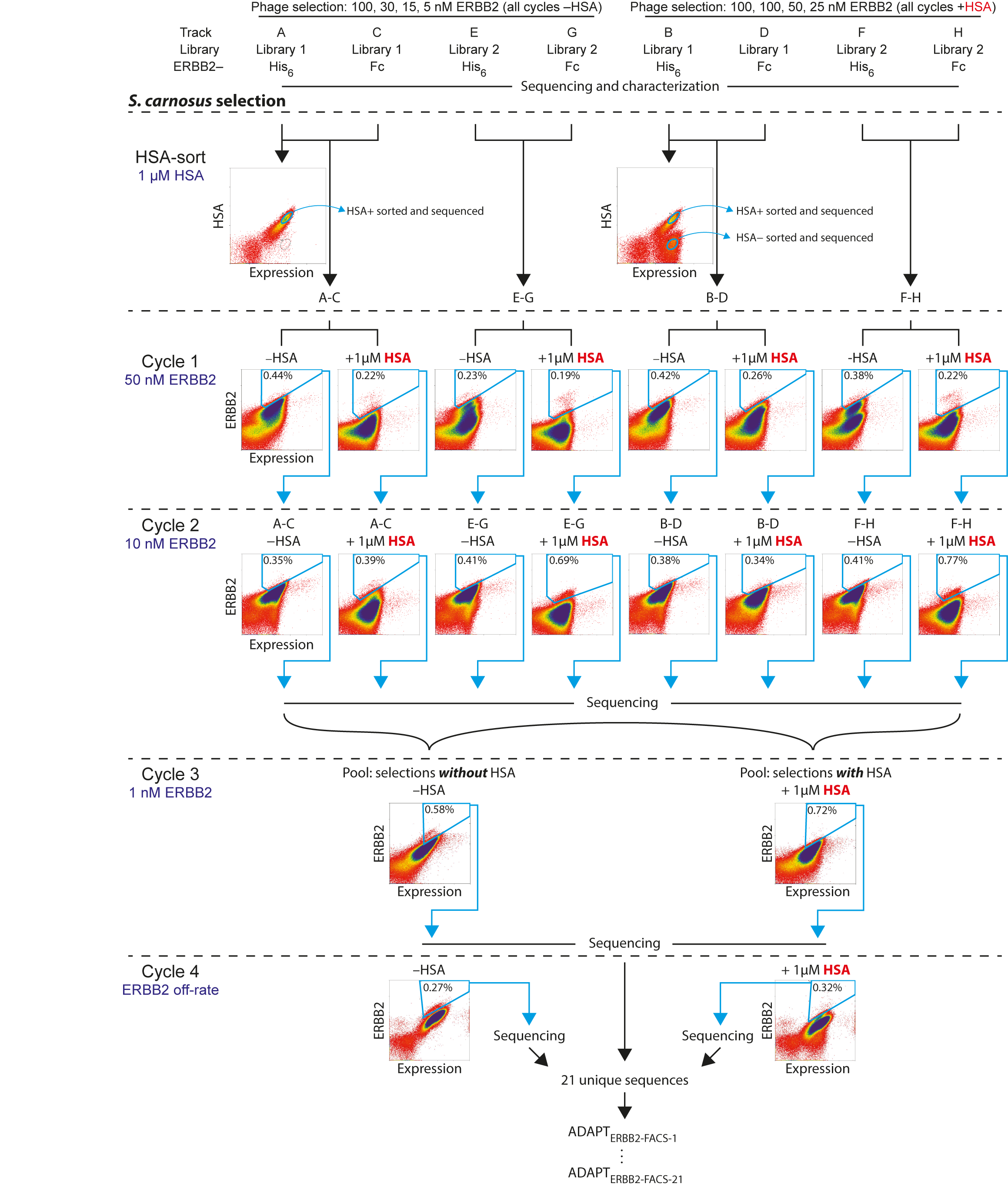

Supplement: Figure S4 — Overview of cell sortings during affinity maturation (A). Eight selection tracks (A–H) from the two phage display libraries (library 1 and 2) where displayed on staphylococcal cells to enable fluorescence-activated cell sorting for ERBB2-binding. Cells from tracks A and B were sorted based on HSA-binding and sequenced before the first cycle of ERBB2-selection. Cells were pooled in four sub-pools based on the previously applied selection strategy (with or without 1 µM unlabelled HSA present) and library design (Library 1 or 2). All pools were selected for ERBB2-binding both with and without excess albumin present in eight sorting experiments for two rounds. After two rounds of sorting, individual clones were sequenced and the outputs from the second cycle were pooled only based on selection strategy (into two sub-pools). DNA sequencing after the last two sorting cycles identified 21 unique sequences. Sort gates and the percentage of analyzed cells that was sorted in each experiment are indicated in each diagram. ERBB2-binding (measured as fluorescence from streptavidin-R-phycoerythrin) is shown on the y-axis and surface expression level (measured as fluorescence from bound IgG conjugated to Alexa Fluor 647) is shown on the x-axis, data are shown using logarithmic scales. Surface expression level was monitored to allow normalization of the ERBB2-binding signal and minimize potential biases from differences in surface expression levels. (TIF) [file pone.0103094.s004.tif]
