# Supplementary material for: Engineering of Bispecific Affinity Proteins with High Affinity for ERBB2 and Adaptable Binding to Albumin
Source: PLoS One. 2014 Aug 4;9(8):e103094. doi: 10.1371/journal.pone.0103094 (PMC4121139; doi:10.1371/journal.pone.0103094)
Supplement: Table S1 — Affinities of selected affinity-matured ADAPTs after phage display. Kinetic parameters are presented as mean values with standard deviation. KD was calculated from kd/ka and the number of replicates is indicated within brackets. Ten selected molecules, ADAPTERBB2-mat1-ADAPTERBB2-mat10, were included in the analysis. (DOCX) [file pone.0103094.s006.docx]

|  | **ERBB2** | | |  | **HSA** | | |  |
| --- | --- | --- | --- | --- | --- | --- | --- | --- |
|  | k_a_  (M^-1^s^-1^) | k_d_  (s^-1^) | K_D_  (nM) |  | k_a_  (M^-1^s^-1^) | k_d_  (s^-1^) | K_D_  (nM) | T_m_ (°C) |
| ADAPT_ERBB2-mat1_ | 2.8 (±0.7)·10^5^ | 6.4 (±1.1)·10^-4^ | 2.3 (n = 7) |  | 1.2 (±0.4)·10^5^ | 2.3 (±0.8)·10^-3^ | 20 (2) | 54 |
| ADAPT_ERBB2-mat2_ | 2.2 (±0.4)·10^5^ | 4.4 (±0.5)·10^-4^ | 2.0 (7) |  | 9.3 (±1.3)·10^4^ | 2.7 (±0.4)·10^-4^ | 2.9 (2) | N.D. |
| ADAPT_ERBB2-mat3_ | 3.2 (±0.4)·10^5^ | 3.3 (±0.5)·10^-4^ | 1.1 (7) |  | 2.0 (±0.1)·10^5^ | 2.2 (±0.3)·10^-4^ | 1.1 (2) | 79 |
| ADAPT_ERBB2-mat4_ | 5.8 (±1.0)·10^5^ | 6.0 (±0.8)·10^-4^ | 1.0 (7) |  | 2.3 (±0.5)·10^5^ | 3.0 (±0.5)·10^-4^ | 1.3 (2) | 69 |
| ADAPT_ERBB2-mat5_ | 3.5 (±0.7)·10^5^ | 1.1 (±0.1)·10^-3^ | 3.1 (7) |  | 1.3 (±0.3)·10^5^ | 4.6 (±0.6)·10^-4^ | 3.7 (2) | 75 |
| ADAPT_ERBB2-mat6_ | 4.0 (±0.8)·10^5^ | 3.6 (±0.4)·10^-4^ | 0.9 (7) |  | 1.9 (±0.1)·10^5^ | 2.4 (±0.4)·10^-4^ | 1.3 (2) | 63 |
| ADAPT_ERBB2-mat7_ | 3.9 (±0.5)·10^5^ | 1.1 (±0.2)·10^-3^ | 2.9 (7) |  | 1.5 (±0.2)·10^5^ | 4.9 (±0.4)·10^-4^ | 3.3 (2) | 67 |
| ADAPT_ERBB2-mat8_ | 2.9 (±0.4)·10^5^ | 2.7 (±0.4)·10^-3^ | 9.4 (7) |  | 1.2 (±0.3)·10^5^ | 2.0 (±0.5)·10^-3^ | 17 (2) | 56 |
| ADAPT_ERBB2-mat9_ | 2.6 (±0.4)·10^5^ | 2.1 (±0.2)·10^-3^ | 8.1 (7) |  | 8.3 (±1.1)·10^4^ | 7.7 (±0.1)·10^-4^ | 9.3 (2) | 54 |
| ADAPT_ERBB2-mat10_ | 2.0 (±0.3)·10^5^ | 7.8 (±0.8)·10^-4^ | 3.9 (7) |  | 6.8 (±0.7)·10^4^ | 3.7 (±0.2)·10^-4^ | 5.5 (2) | N.D. |
